# Reproduction Is Associated with a Tissue-Dependent Reduction of Oxidative Stress in Eusocial Female Damaraland Mole-Rats (*Fukomys damarensis*)

**DOI:** 10.1371/journal.pone.0103286

**Published:** 2014-07-28

**Authors:** Christina M. Schmidt, Jonathan D. Blount, Nigel C. Bennett

**Affiliations:** 1 Department of Zoology and Entomology, University of Pretoria, Pretoria, Gauteng, South Africa; 2 Centre for Ecology and Conservation, College of Life and Environmental Sciences, University of Exeter, Penryn Campus, Penryn, Cornwall, United Kingdom; University of Cincinnati, United States of America

## Abstract

Oxidative stress has been implicated as both a physiological cost of reproduction and a driving force on an animal's lifespan. Since increased reproductive effort is generally linked with a reduction in survival, it has been proposed that oxidative stress may influence this relationship. Support for this hypothesis is inconsistent, but this may, in part, be due to the type of tissues that have been analyzed. In Damaraland mole-rats the sole reproducing female in the colony is also the longest lived. Therefore, if oxidative stress does impact the trade-off between reproduction and survival in general, this species may possess some form of enhanced defense. We assessed this relationship by comparing markers of oxidative damage (malondialdehyde, MDA; protein carbonyls, PC) and antioxidants (total antioxidant capacity, TAC; superoxide dismutase, SOD) in various tissues including plasma, erythrocytes, heart, liver, kidney and skeletal muscle between wild-caught reproductive and non-reproductive female Damaraland mole-rats. Reproductive females exhibited significantly lower levels of PC across all tissues, and lower levels of MDA in heart, kidney and liver relative to non-reproductive females. Levels of TAC and SOD did not differ significantly according to reproductive state. The reduction in oxidative damage in breeding females may be attributable to the unusual social structure of this species, as similar relationships have been observed between reproductive and non-reproductive eusocial insects.

## Introduction

Investment in the production of offspring is customarily linked to compromised survival, as prolific reproduction tends to be coupled with a relatively shorter life span [1], [2], [3]. This relationship has been classically characterized as being driven by the diversion of resources from self-maintenance towards reproduction, however, attention has been more recently turned to the investigation of physiological costs of reproduction that impair functionality of one or more physiological processes[4]. Such costs may result from the reduction of resources available for self-maintenance, but they may also be direct effects of the process of reproduction, itself [4], [5].The production of offspring is associated with a myriad of physiological adjustments, and it has been frequently proposed that oxidative stress resulting from such changes is one such cost of reproduction.

Oxidative stress arises when the production of reactive oxygen species (ROS), which damage proteins, lipids and DNA, exceeds the capacity of antioxidants and repair mechanisms to prevent or mitigate ROS damage [6], [7], [8]. It has been proposed that elevated reproductive effort should increase an animal's vulnerability to oxidative stress [9], [10], [11], and this has been demonstrated in some bird and reptile species [9], [12], [13], [14], [15], [16]. In eutherian mammals, ROS production is elevated, in part, by mitochondrial activity of the placenta[17], [18], and during gestation sows, humans and sheep exhibit an increase in oxidative damage along with a reduction in antioxidant capacity[19], [20], [21], [22], [23](but see[24]).Oxidative damage also increases with number or mass of offspring produced in sheep, mice and Eastern chipmunks (*Tamias striatus*) [25], [26], [27], [28].

Oxidative stress is also associated with numerous pathologies (see [29]), and the accumulation of oxidative damage has long been considered a mechanism by which animals age [30], [31]. Across several animal taxa there is a negative correlation between maximum lifespan and endogenous levels of tissue antioxidants [32] and long-lived species exhibit low rates of ROS production relative to oxygen consumption near DNA paired with high rates of DNA repair [33]. Thus, oxidative stress that accompanies the production of offspring has frequently been suggested to be the physiological link between reproduction and lifespan.

It is important to note that many investigations into the relationship between reproduction and oxidative stress have measured biomarkers of oxidative stress only in serum or plasma samples (see [34]). While this type of sampling is more practical for field and longitudinal studies, individual tissue types may vary in the level of oxidative damage exhibited at a given time within an animal (see [35]). Reproductive female Sprague-Dawley rats have higher levels of lipid oxidation in lung, uterus, brain, kidney and thymus, but not in the liver or spleen, relative to non-reproductive females [36] and reproductive female mice have less protein and lipid oxidation, higher concentration of antioxidants in the liver, and less lipid oxidation in skeletal muscle than do non-reproductive females (this difference was not detected in the serum; [26]). In bank voles (*Myodes gareolus*) lipid oxidation was lower in skeletal muscle and kidney and protein oxidation tended to be lower in the heart of reproductive females relative to non-reproductive females, whereas there was no difference in levels of oxidative damage in the liver between reproductive and non-reproductive females [37]. Thus, serum and plasma measurements by themselves may not accurately portray the balance between ROS production and antioxidant activity.

Upon investigating oxidative stress in various tissues, the aforementioned studies also reveal that in some species reproducing females are experiencing less oxidative damage than those that do not reproduce. This relationship has been observed in social insect species (see [38]) and, interestingly, these reproductive females, or queens, live much longer than non-reproductive members of the colony [39], [40], [41]. In mammals, social species of African mole-rats (Bathyergidae) exhibit both a reproductive hierarchy and extended lifespan of reproducing females akin to that of social insects [42], [43], [44], [45], [46], [47], [48], and Damaraland mole-rats (*Fukomys damarensis*), which, along with naked mole-rats (*Heterocephalus glaber*), are argued to be the only truly eusocial mammals[43]. Like social bees, ants and termites, there is only one female in a Damaraland mole-rat colony that reproduces, all other female colony members are reproductively suppressed [44] and the reproductive female lives longer than her non-reproductive counterparts [45]. Upon separation from the reproductive female, a reproductively-suppressed female commences ovulation and thus can feasibly begin to reproduce [49], suggesting that reproductive status is not genetically regulated. It has been suggested that reproductive female Ansell's mole-rats possess stronger defenses against oxidative damage relative to non-reproductive females [50], however, to date little is known about how reproduction affects susceptibility to oxidative stress in this uniquely social group of mammals.

If reproductive mole-rats experience less oxidative stress than their non-reproductive cohorts, this may serve as a mechanism that underlies their extended lifespan. To determine this, we measured oxidative stress in several different tissues of reproductive and non-reproductive female Damaraland mole-rats, predicting that reproductive females have less oxidative damage and better antioxidant defense relative to their non-reproductive cohorts, and that there will be variation between tissue types as to how this difference in relation to reproduction is manifested. Although it has been suggested that a comparison between reproductive and non-reproductive females can be confounded by a female's ability to adjust reproductive investment [51], any degree of reproductive investment will result in an elevation of metabolic rate relative to non-reproductive females [34]. Additionally, many potential problems may accompany suggested means for controlling and manipulating reproductive investment, such as the impact of physiological constraints that are unaffected by experimental manipulation (see [34]). Thus, our approach is argued to be not only valid, but ideal for assessing oxidative stress as a potential cost of reproduction [34].

## Materials and Methods

### Ethics Statement

Research protocols were approved by the animal ethics committee at the University of Pretoria and complied with their guidelines for animal research (protocol number EC008-12).

### Animals

We selected 9 reproductive and 14 non-reproductive females from colonies at the University of Pretoria that had been recently collected (following [52]) from the area surrounding the towns of Hotazel and Blackrock, Northern Cape Province, South Africa. Reproductive females were initially differentiated from non-reproductive females by their swollen teats or perforate vaginas [44] and later confirmed by the presence of placental scars. The number of previous reproductive bouts could not be determined. As these were wild-caught animals, exact age was not known, but they were all adult based on body mass measurements. Reproductive and non-reproductive females were kept together with other members of their colony to maintain their reproductive status, and were housed in large plastic boxes lined with wood shavings and paper nesting materials. We provided all animals with *ad libitum* access to a combination of sweet potatoes, carrots, apples and gemsbok squash.

### Sample Collection

On 3–4 July 2012, we euthanized animals via decapitation and immediately collected about 1 mL of blood in heparinized tubes. We then centrifuged the sample for 10 min at 1,000×g, drew off the plasma, transferred it to plastic tubes, and stored plasma and erythrocytes in a −80 freezer until time of analysis, which was within a period of 40 days. We removed the heart, left kidney, a section of the left median lobe of the liver, and the vastus lateralis of the left leg (herein, skeletal muscle) immediately following decapitation and snap froze them in liquid nitrogen. We homogenized liver, heart, skeletal muscle and kidney on ice in 10% weight per volume 100 mM HEPES (N-2 hydroxyethylpiperazine-N′-2-ethanesulfonic acid) buffer solution for 1 (liver and kidney) or 2 (heart and skeletal muscle) minutes on an Ultra Turrax T18 Basic Homogenizer (IKA, Staufen, Germany), and stored all homogenates in a -80 freezer until time of analysis.

### Analyses of Oxidative Stress

Oxidative stress represents an imbalance between ROS production, resulting in oxidative damage, and antioxidant defenses or repair mechanisms, and is thus more accurately characterized by including a range of assays of these damage and protection mechanisms [34], [53]. We quantified oxidative damage by concentrations of malondialdehyde (MDA), a marker of lipid peroxidation [54], and protein carbonyls (PC), which indicates protein oxidation [55]. We assessed antioxidant levels as superoxide dismutase (SOD) activity as well as total antioxidant capacity (TAC). The specific tissues used for each of these assays are described below.

### MDA

We measured concentrations of MDA in all tissue homogenates (i.e. liver, kidney, skeletal muscle, heart) and in plasma using high performance liquid chromatography (HPLC) as described by Nussey et al. [24]. We prepared samples following Nussey et al.[24] and injected 20 µL of sample into a Dionex HPLC system (Dionex Corporation, California, USA) fitted with a 5 µm ODS guard column and a Hewlett-Packard Hypersil 5 µ ODS 100×4.6 mm column maintained at 37°C. The mobile phase was methanol-buffer (40∶60, v/v; 50 mM anhydrous solution of potassium monobasic phosphate at pH 6.8), running isocratically over 3.5 min at a flow rate of 1 mL min^−1^. We collected data using a fluorescence detector (RF2000; Dionex) set at 515 nm (excitation) and 553 nm (emission). For calibration, we prepared a standard curve using a TEP stock solution (5 µM in 40% ethanol) serially diluted using 40% ethanol. Results are expressed as nmol MDA per g tissue or ml plasma.

### PC

We measured concentrations of PC in all tissue homogenates (i.e. liver, kidney, skeletal muscle, heart) and in plasma. Oxidation or oxidative cleavage of proteins results in the production of carbonyl groups [55], which covalently reacts with 2,4-dinitrophenylhydrazine (DNPH) to form 2,4-dinitrophenyl (DNP) hydrazone. DNP can then be detected via spectrophotometry at a wavelength of 370nm [56]. We measured PC concentration using a commercially available kit (Protein Carbonyl Assay Kit, Cayman Chemical Co., Ann Arbor, MI, USA), reading absorbances using a Spectramax M2 plate reader (Molecular Devices Corp., Sunnyvale, CA, USA). We then quantified the protein content of each sample from a bovine serum albumin (BSA) standard curve. Results are expressed as nmol per mg protein.

### SOD

We measured SOD activity in all tissue homogenates (i.e. liver, kidney, skeletal muscle, heart) and in erythrocytes. SOD is an enzymatic antioxidant that catalyses the dismutation of superoxide anions to oxygen and hydrogen peroxide [57]. We measured SOD content with a commercially available kit (Superoxide Dismutase Assay Kit, Cayman Chemical Co., Ann Arbor, MI, USA) that measures the percentage of superoxide radicals that undergo dismutation in a given sample. Absorbances were read at 440 nm using a Spectramax M2 plate reader (Molecular Devices Corp., Sunnyvale,CA, USA). Erythrocyte lysate was used instead of plasma for this assay only. Results are expressed as units of SOD activity per mg tissue.

### TAC

We measured TAC in homogenates of liver, kidney and heart, and in plasma. We did not measure TAC in skeletal muscle because intracellular antioxidant defenses (e.g. SOD) are likely to be more important in this tissue type. TAC was quantified using a commercially available kit (Antioxidant Assay Kit, Cayman Chemical Co., Ann Arbor, MI, USA) which measures the oxidation of ABTS (2,2′-Azino-di-[3-ethybenzthiazoline sulphonate]) by metmyoglobin, which is inhibited by non-enzymatic antioxidants contained in the sample. Oxidized ABTS can then be detected via spectrophotometry at a wavelength of 740 nm. The capacity of antioxidants in the sample to inhibit oxidation of ABTS is compared with the capacity of known concentrations of Trolox, and results are expressed as nmol of Trolox equivalents per g tissue or ml plasma.

### Statistical Analyses

Data were examined for normality, homoscedasticity, and outliers. For MDA and SOD, data were log_10_-transformed to improve the approximation of normality. Individual markers of oxidative damage and antioxidant defense may vary in their association with reproductive state, and such relationships are likely to differ amongst tissues [58]. Therefore, we assessed whether each marker of oxidative damage or antioxidant defense in turn differed between reproductive and non-reproductive females, using General Linear Mixed Models (GLMM) with reproductive state and tissue (and their interaction) as fixed factors, and with individual identity and colony membership included as random factors. Degrees of freedom were calculated using Satterthwaite's correction. Models were developed by backward elimination of non-significant terms (where *P*>0.05) starting with the reproductive state x tissue interaction term. Significant reproductive state x tissue interactions were followed by post-hoc GLMMs for each tissue in turn, including reproductive state as a fixed factor and colony membership as a random factor. In the absence of any association with reproductive state, we were not interested in differences in oxidative damage or antioxidant defenses amongst tissues *per se*. Such differences are inevitable given the wide variety of tissues included in this study. Therefore, any significant main effect of tissue was not followed by post-hoc tests. Not all tissue samples were available for each individual and each assay, resulting in a variation in sample size between tissue types (Table S1). All analyses were performed using Genstat (16^th^ edition) (VSN International Ltd., Hemel Hempstead, UK). Results are reported as means ± s.e.

## Results

**Figure 1:**
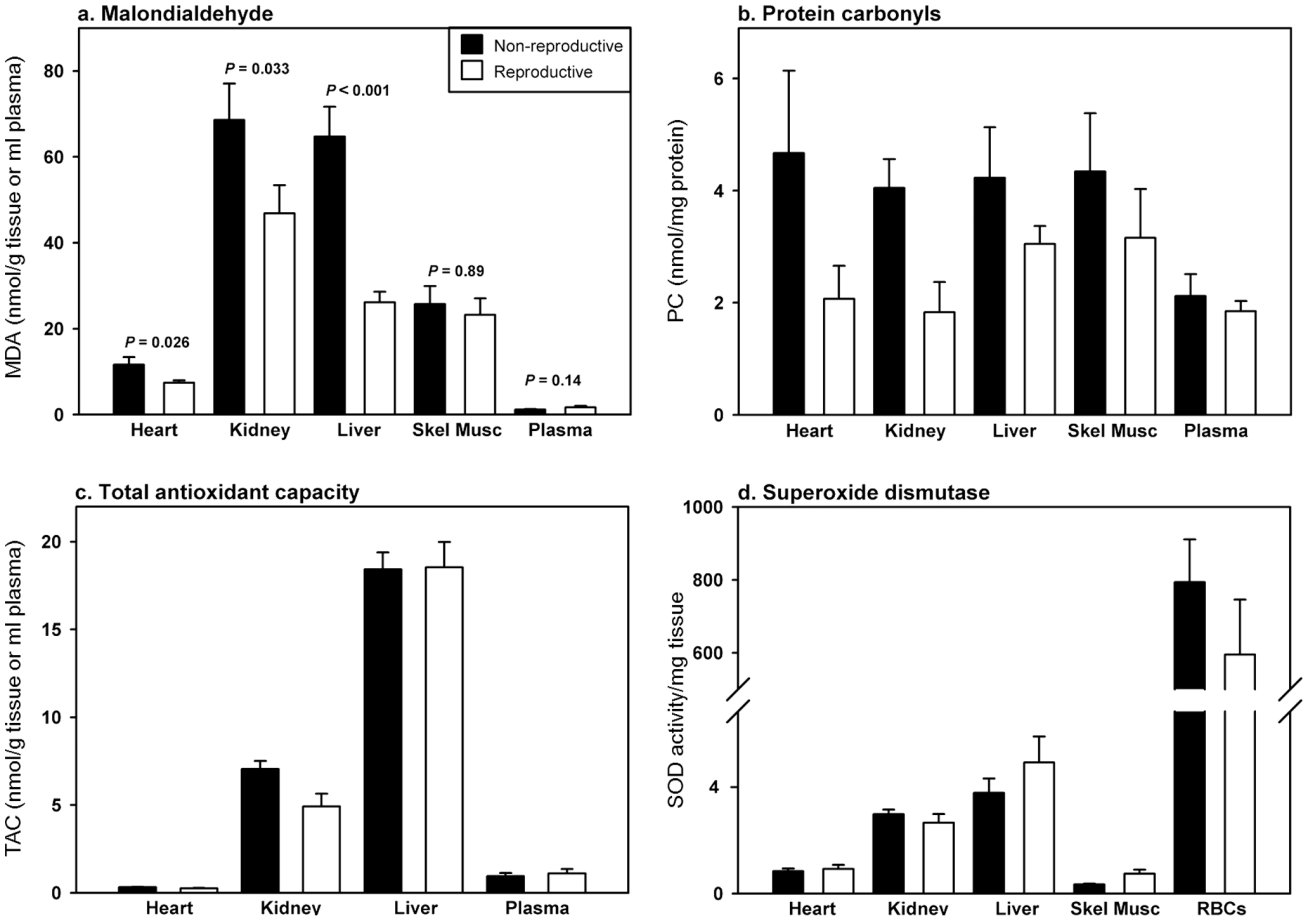
Oxidative stress markers of different tissue types for reproductive and non-reproductive females. Concentrations of markers of oxidative damage (a. malondialdehyde and b. protein carbonyls) and antioxidant activity (c. superoxide dismutase and d. total antioxidant capacity) in the heart, kidney, liver, skeletal muscle (skel musc) and plasma or erythrocytes (RBCs) of non-reproductive (black boxes) and reproductive (white boxes) adult female Damaraland mole-rats.

Levels of MDA were significantly decreased in reproductive compared to non-reproductive females, although this differed amongst tissue types. Post-hoc analyses revealed that MDA was significantly lower in the heart, kidney and liver in reproductive compared to non-reproductive females, but did not differ significantly according to reproductive state in skeletal muscle or plasma (Fig. 1a and Table 1). Levels of PC were significantly decreased in reproductive compared to non-reproductive females, and this was apparent across all tissues (Fig. 1b and Table 1). SOD activity did not differ significantly according to reproductive state, but varied markedly amongst tissues (Fig. 1c and Table 1). Levels of TAC showed a similar pattern, not differing significantly between reproductive and non-reproductive females, but varying markedly amongst tissues (Fig. 1d and Table 1).

**Table 1:** Variation in markers of oxidative damage (MDA and PC) and antioxidant defence (TAC and SOD) in relation to reproductive state and tissue.

Response	Explanatory	*F*	d.f.	*P*
MDA	Reproductive state	17.07	1,17.9	**<0.001**
	Tissue	298.31	4,81.2	**<0.001**
	Reproductive state x tissue	5.54	4,81.5	**<0.001**
PC	Reproductive state	10.40	1,16.6	**0.005**
	Tissue	2.04	4,65.8	0.10
	Reproductive state x tissue	0.76	4,60.8	0.55
SOD	Reproductive state	0.23	1,21.5	0.64
	Tissue	212.19	4,78.9	**<0.001**
	Reproductive state x tissue	0.48	4,74.2	0.75
TAC	Reproductive state	1.14	1,16.0	0.30
	Tissue	349.14	3,62.3	**<0.001**
	Reproductive state x tissue	1.54	3,58.9	0.21

Results are from General Linear Mixed Models including individual identity and colony membership as random factors. See Methods for details. Significant *P* values are shown in bold.

## Discussion

Lower concentrations of both markers of oxidative damage observed in reproductive females suggest that either these females produced less ROS than non-reproductive females, or that antioxidant defenses were more active in reproductive females. Mitochondrial uncoupling can result in reduced ROS production, although studies of mice have indicated that expression of the genes that code for uncoupling proteins is reduced or unchanged in breeders compared with non-breeders [5]. Therefore, mitochondrial uncoupling seems an unlikely general explanation for reduced levels of oxidative damage in various tissues of female Damaraland mole-rats. However, since we did not explicitly quantify ROS production and scavenging, we cannot rule out the possibility that reproductive females may have produced less ROS than non-reproductive females. Elevated ROS production has been observed in other mammal species during reproduction, and a variety of taxa, including sheep, mice, Pacific oysters (*Crassostrea gigas*) and painted dragon lizards (*Ctenophorus pictus*), all exhibited increased expression of antioxidants during reproduction [16], [59], [60], [61], [62]. Thus, the difference in oxidative damage observed in Damaraland mole-rats seems more likely to be attributable to variation in antioxidant activity. The absence of variation in TAC and SOD concentrations between reproductive and non-reproductive female Damaraland mole-rats intimates that either antioxidant defenses matched or exceeded ROS production during reproduction, or that other antioxidant systems play a more substantial role in this process.

Several physiological adjustments accompany a non-reproductive Damaraland mole-rat's transition to reproductive status, including increased body length, brain volume, reproductive hormone concentrations and pituitary sensitivity [52], [63], [64], [65], [66]. It is possible that any combination of these adjustments may reflect or drive a decreased susceptibility to oxidative stress, and this effect could be enhanced by physiological adjustments associated with gestation and lactation, themselves. An example of this is found in honey bees (*Apis mellifera*), in which reproduction is associated with an increased production of the protein vitellogenin, which defends against oxidative stress [67], [68]. Some vitellogenin sequences are conserved amongst vertebrates, insects and nematodes [69], and vitellogenin is thought to be related to mammalian low-density lipoproteins [70], [71]. However, to date this potential relationship in mammals has yet to be explored.

Additionally, the reduction of oxidative stress found in reproductive Damaraland mole-rats and other species may be indicative of hormetic response, in which exposure to ROS associated with reproduction or becoming reproductive primes the female's system to defend against long-term oxidative challenges [72], [73], [74], [75], [76]. Exercise also factors into the hormetic framework; whereas vigorous and intermittent physical activity promotes oxidative stress, regular and moderate exercise reduces oxidative stress [77], [78]. This is noteworthy, as non-reproductive Damaraland mole-rats, along with other social mole-rat species (naked mole-rats and common mole-rats (*Cryptomys hottentotus*)),spend much more time sleeping than the reproductive female [44], [79], [80].Thus, the difference in physical activity between reproductive and non-reproductive females may play a role in differentiating oxidative stress characteristics of these two groups.

Levels of PC were reduced in all tissues of reproductive females, however, levels of MDA showed different patterns amongst tissues in relation to reproduction; MDA was reduced in heart, kidney and liver, but did not differ in skeletal muscle or plasma. This observation underscores the importance of assessing oxidative stress in more than one tissue in order to obtain a more thorough depiction of oxidative stress dynamics within an animal [34]. Our findings support observed differences in biomarkers of oxidative stress between tissues in other species [35], [81], with some tissues being more susceptible, such as the liver relative to skeletal muscle [77], which is likely attributed to variation in metabolic rate between tissues [82], [83]. For example, about 60% of resting energetic expenditure is attributed to metabolic activity of the brain, liver and kidneys in humans [83]. The composition of the different tissues may also drive variation in susceptibility. Cells that comprise the parenchyma of kidney and liver are constantly dividing, whereas those of the heart and skeletal muscle are post-mitotic, and it is generally thought that age-associated changes are stronger and more widespread in the latter cell type [84].

While several exceptions to the oxidative stress theory of aging have emerged (see [74]), our results do not negate the idea that accumulated oxidative damage is related to aging. Instead, our results show that less oxidative damage is present in reproductive female Damaraland mole-rats, which live longer than non-reproductive females. This may be, in part, attributable to our analysis of multiple tissues. Given our results, the question arises as to whether oxidative stress can be considered to be a cost of reproduction in Damaraland mole-rats, and, indeed, whether this species experiences any physiological costs of reproduction. Our results correlate reproduction, oxidative stress and lifespan, but it is important to quantify oxidative stress relative to survival rate, and to control for, or experimentally manipulate, reproductive effort before strong conclusions can be made regarding this relationship [85].

There is evidence that lifespan is not traded off against reproduction in some social insect species [41], [68], [86], [87], [88] and in Ansell's mole-rats it has been proposed that, given no difference in activity or intrinsic quality between reproductive and non-reproductive individuals, reproduction may drive increased longevity in breeding females [46]. Since the reproductive success of a eusocial colony is almost solely dependent on the condition of the queen, Damaraland mole-rats and other eusocial species may have acquired adaptations to ensure or enhance survival that are expressed when a female obtains dominance and commences breeding. Ascension to dominant status is largely driven by environmental stimuli [49], [66], however, it is possible that an individual's capacity to defend against oxidative damage may also influence likelihood of successfully attaining reproductive status.

Thus, in this and other eusocial species, oxidative stress may serve as a potential link between reproduction and lifespan which could function as a basis for prospective avenues for investigating the evolution of sociality as well as life-history traits and reproductive strategies.

## Supporting Information

Table S1 **Data used for the analyses of markers of oxidative damage and antioxidant defence in various tissues in relation to reproductive state.**
(DOCX)

## References

[1] Williams GC (1966) Natural selection, the costs of reproduction, and a refinement of Lack's principle. American Naturalist: 687–690.

[2] Stearns SC (1992) The evolution of life histories. Oxford: Oxford University Press.

[3] Roff DA (1992) The evolution of life histories: theory and analysis. New York: Chapman and Hall.

[4] Harshman LG, Zera AJ (2007) The cost of reproduction: the devil in the details. Trends in Ecology & Evolution 22: 80–86. 17056152 10.1016/j.tree.2006.10.008

[5] Speakman JR (2008) The physiological costs of reproduction in small mammals. Philosophical Transactions of the Royal Society B-Biological Sciences 363: 375–398. 10.1098/rstb.2007.2145 PMC2606756 17686735

[6] Sies H (1991) Oxidative Stress II. Oxidants and Antioxidants. London: Academic Press.

[7] Finkel T, Holbrook NJ (2000) Oxidants, oxidative stress and the biology of aging. Nature 408: 239–247. 11089981 10.1038/35041687

[8] Costantini D (2008) Oxidative stress in ecology and evolution: lessons from avian studies. Ecology Letters 11: 1238–1251. 18803642 10.1111/j.1461-0248.2008.01246.x

[9] Alonso-Alvarez C, Bertrand S, Devevey G, Prost J, Faivre B, et al (2004) Increased susceptibility to oxidative stress as a proximate cost of reproduction. Ecology Letters 7: 363–368.

[10] Salmon AB, Marx DB, Harshman LG (2001) A cost of reproduction in Drosophila melanogaster: Stress susceptibility. Evolution 55: 1600–1608. 11580019 10.1111/j.0014-3820.2001.tb00679.x

[11] Wang Y, Salmon AB, Harshman LG (2001) A cost of reproduction: oxidative stress susceptibility is associated with increased egg production in Drosophila melanogaster. Experimental Gerontology 36: 1349–1359. 11602209 10.1016/s0531-5565(01)00095-x

[12] Alonso-Alvarez C, Bertrand S, Devevey G, Prost J, Faivre B, et al (2006) An experimental manipulation of life-history trajectories and resistance to oxidative stress. Evolution 60: 1913–1924. 17089975

[13] Bertrand S, Alonso-Alvarez C, Devevey G, Faivre B, Prost J, et al (2006) Carotenoids modulate the trade-off between egg production and resistance to oxidative stress in zebra finches. Oecologia 147: 576–584. 16341888 10.1007/s00442-005-0317-8

[14] Wiersma P, Selman C, Speakman JR, Verhulst S (2004) Birds sacrifice oxidative protection for reproduction. Proceedings of the Royal Society of London Series B: Biological Sciences 271: S360–S363. 15504018 10.1098/rsbl.2004.0171 PMC1810045

[15] Christe P, Glaizot O, Strepparava N, Devevey G, Fumagalli L (2012) Twofold cost of reproduction: an increase in parental effort leads to higher malarial parasitaemia and to a decrease in resistance to oxidative stress. Proceedings of the Royal Society B-Biological Sciences 279: 1142–1149. 10.1098/rspb.2011.1546 PMC3267143 21920974

[16] Olsson M, Healey M, Perrin C, Wilson M, Tobler M (2012) Sex-specific SOD levels and DNA damage in painted dragon lizards (*Ctenophorus pictus*). Oecologia 170: 917–924. 22700064 10.1007/s00442-012-2383-z

[17] Casanueva E, Viteri FE (2003) Iron and oxidative stress in pregnancy. Journal of Nutrition 133: 1700S–1708S. 12730487 10.1093/jn/133.5.1700S

[18] Myatt L, Cui XL (2004) Oxidative stress in the placenta. Histochemistry and Cell Biology 122: 369–382. 15248072 10.1007/s00418-004-0677-x

[19] Fainaru O, Almog B, Pinchuk I, Kupferminc MJ, Lichtenberg D, et al (2002) Active labour is associated with increased oxidisibility of serum lipids ex vivo. BJOG: An International Journal of Obstetrics & Gynaecology 109: 938–941. 12197375 10.1111/j.1471-0528.2002.01494.x

[20] Garrel C, Fowler PA, Al-Gubory KH (2010) Developmental changes in antioxidant enzymatic defenses against oxidative stress in sheep placentomes. Journal of Endocrinology 205: 107–116. 20097690 10.1677/JOE-09-0362

[21] Berchieri-Ronchi CB, Kim SW, Zhao Y, Correa CR, Yeum KJ, et al (2011) Oxidative stress status of highly prolific sows during gestation and lactation. Animal 5: 1774–1779. 22440418 10.1017/S1751731111000772

[22] Makedou K, Kourtis A, Gkiomisi A, Toulis KA, Mouzaki M, et al (2011) Oxidized low-density lipoprotein and adiponectin levels in pregnancy. Gynecological Endocrinology 27: 1070–1073. 21504339 10.3109/09513590.2011.569793

[23] Mohebbi-Fani M, Mirzaei A, Nazifi S, Shabbooie Z (2012) Changes of vitamins A, E, and C and lipid peroxidation status of breeding and pregnant sheep during dry seasons on medium-to-low quality forages. Tropical Animal Health and Production 44: 259–265. 22083269 10.1007/s11250-011-0012-1

[24] Nussey DH, Pemberton JM, Pilkington JG, Blount JD (2009) Life history correlates of oxidative damage in a free-living mammal population. Functional Ecology 23: 809–817.

[25] Bergeron P, Careau V, Humphries MM, Réale D, Speakman JR, et al (2011) The energetic and oxidative costs of reproduction in a free-ranging rodent. Functional Ecology 25: 1063–1071.

[26] Garratt M, Vasilaki A, Stockley P, McArdle F, Jackson M, et al (2011) Is oxidative stress a physiological cost of reproduction? An experimental test in house mice. Proceedings of the Royal Society B-Biological Sciences 278: 1098–1106. 10.1098/rspb.2010.1818 PMC3049035 20926440

[27] Gur S, Turk G, Demirci E, Yuce A, Sonmez M, et al (2011) Effect of pregnancy and foetal number on diameter of corpus luteum, maternal progesterone concentration and oxidant/antioxidant balance in ewes. Reproduction in Domestic Animals 46: 289–295. 20565696 10.1111/j.1439-0531.2010.01660.x

[28] Stier A, Reichert S, Massemin S, Bize P, Criscuolo F (2012) Constraint and cost of oxidative stress on reproduction: correlative evidence in laboratory mice and review of the literature. Frontiers in Zoology 9. 10.1186/1742-9994-9-37 PMC3551645 23268929

[29] Abuja PM, Albertini R (2001) Methods for monitoring oxidative stress, lipid peroxidation and oxidation resistance of lipoproteins. Clinica Chimica Acta 306: 1–17. 10.1016/s0009-8981(01)00393-x 11282089

[30] Harman D (1956) Aging - a theory based on free-radical and radiation-chemistry. Journals of Gerontology 11: 298–300. 13332224 10.1093/geronj/11.3.298

[31] Monaghan P, Charmantier A, Nussey DH, Ricklefs RE (2008) The evolutionary ecology of senescence. Functional Ecology 22: 371–378.

[32] Pamplona R, Costantini D (2011) Molecular and structural antioxidant defenses against oxidative stress in animals. American Journal of Physiology-Regulatory Integrative and Comparative Physiology 301: R843–R863. 10.1152/ajpregu.00034.2011 21775650

[33] Perez-Campo R, Lopez-Torres M, Cadenas S, Rojas C, Barja G (1998) The rate of free radical production as a determinant of the rate of aging: evidence from the comparative approach. Journal of Comparative Physiology B-Biochemical Systemic and Environmental Physiology 168: 149–158. 10.1007/s003600050131 9591361

[34] Speakman JR, Garratt M (2014) Oxidative stress as a cost of reproduction: beyond the simplistic trade-off model. Bioessays 36: 93–106. 24285005 10.1002/bies.201300108

[35] Lopez-Torres M, Perez-Campo R, Cadenas S, Rojas C, Barja G (1993) A comparative-study of free-radicals in vertebrates. 2. Nonenzymatic antioxidants and oxidative stress. Comparative Biochemistry and Physiology B-Biochemistry & Molecular Biology 105: 757–763. 10.1016/0305-0491(93)90117-n 8365120

[36] Sainz RM, Reiter RJ, Mayo JC, Cabrera J, Tan DX, et al (2000) Changes in lipid peroxidation during pregnancy and after delivery in rats: effect of pinealectomy. Journal of Reproduction and Fertility 119: 143–149. 10864824 10.1530/jrf.0.1190143

[37] Oldakowski L, Piotrowska Z, Chrzascik KM, Sadowska ET, Koteja P, et al (2012) Is reproduction costly? No increase of oxidative damage in breeding bank voles. Journal of Experimental Biology 215: 1799–1805. 22573758 10.1242/jeb.068452

[38] Parker JD (2010) What are social insects telling us about aging? Myrmecological News 13: 103–110.

[39] Keller L (1998) Queen lifespan and colony characteristics in ants and termites. Insectes Sociaux 45: 235–246.

[40] Keller L, Genoud M (1997) Extraordinary lifespans in ants: a test of evolutionary theories of aging. Nature 389: 958–960.

[41] Remolina SC, Hughes KA (2008) Evolution and mechanisms of long life and high fertility in queen honey bees. Age 30: 177–185. 19424867 10.1007/s11357-008-9061-4 PMC2527632

[42] Jarvis JUM (1981) Eusociality in a mammal - cooperative breeding in naked mole-rat colonies. Science 212: 571–573. 7209555 10.1126/science.7209555

[43] Jarvis JUM, Bennett NC (1993) Eusociality has evolved independently in 2 Genera of Bathyergid mole-Rats - but occurs in no other subterranean mammal. Behavioral Ecology and Sociobiology 33: 253–260.

[44] Bennett NC, Faulkes CG (2000) African Mole-rats: Ecology and Eusociality. Cambridge: Cambridge University Press.

[45] Schmidt CM, Jarvis JUM, Bennett NC (2013) The long-lived queen: reproduction and longevity in the eusocial Damaraland mole-rat (*Fukomys damarensis*). African Zoology 48: 193–196.

[46] Dammann P, Burda H (2006) Sexual activity and reproduction delay aging in a mammal. Current Biology 16: R117–R118. 16488857 10.1016/j.cub.2006.02.012

[47] Dammann P, Sumbera R, Massmann C, Scherag A, Burda H (2011) Extended longevity of reproductives appears to be common in Fukomys mole-rats (Rodentia, Bathyergidae). Plos One 6: e18757. 21533255 10.1371/journal.pone.0018757 PMC3076438

[48] Schmidt CM, Jarvis JUM, Bennett NC (2013) The long-lived queen: reproduction and longevity in female eusocial Damaraland mole-rats (*Fukomys damarensis*). African Zoology 48: 193–196.

[49] Molteno AJ, Bennett NC (2000) Anovulation in non-reproductive female Damaraland mole-rats (*Cryptomys damarensis*). Journal of Reproduction and Fertility 119: 35–41. 10864811 10.1530/jrf.0.1190035

[50] Dammann P, Sell DR, Begall S, Strauch C, Monnier VM (2012) Advanced glycation end-products as markers of aging and longevity in the long-lived Ansell's mole-rat (*Fukomys anselli*). Journals of Gerontology Series a-Biological Sciences and Medical Sciences 67: 573–+. 10.1093/gerona/glr208 PMC3348492 22156473

[51] Metcalfe NB, Monaghan P (2013) Does reproduction cause oxidative stress? An open question. Trends in Ecology & Evolution 28: 347–350. 23485157 10.1016/j.tree.2013.01.015

[52] Young AJ, Bennett NC (2010) Morphological divergence of breeders and helpers in wild Damaraland mole-rat societies. Evolution 64: 3190–3197. 20561049 10.1111/j.1558-5646.2010.01066.x

[53] Selman C, Blount JD, Nussey DH, Speakman JR (2012) Oxidative damage, aging, and life-history evolution: where now? Trends in Ecology & Evolution 27: 570–577. 22789512 10.1016/j.tree.2012.06.006

[54] Monaghan P, Metcalfe NB, Torres R (2009) Oxidative stress as a mediator of life history trade-offs: mechanisms, measurements and interpretation. Ecology Letters 12: 75–92. 19016828 10.1111/j.1461-0248.2008.01258.x

[55] Dalle-Donne I, Rossi R, Giustarini D, Milzani A, Colombo R (2003) Protein carbonyl groups as biomarkers of oxidative stress. Clinica Chimica Acta 329: 23–38. 10.1016/s0009-8981(03)00003-2 12589963

[56] Levine RL, Garland D, Oliver CN, Amici A, Climent I, et al (1990) Determination of carbonyl content in oxidatively modified proteins. Methods in Enzymology 186: 464–478. 1978225 10.1016/0076-6879(90)86141-h

[57] Naito Y, Lee M-C-i, Kato Y, Nagai R, Yonei Y (2010) Oxidative stress markers. Anti-Aging Medicine 7: 36–44.

[58] Flores EE, Stevens M, Moore AJ, Blount JD (2013) Diet, development and the optimization of warning signals in post-metamorphic green and black poison frogs. Functional Ecology 27: 816–829.

[59] Yant LJ, Ran QT, Rao L, Van Remmen H, Shibatani T, et al (2003) The selenoprotein GPX4 is essential for mouse development and protects from radiation and oxidative damage insults. Free Radical Biology and Medicine 34: 496–502. 12566075 10.1016/s0891-5849(02)01360-6

[60] Aurousseau B, Gruffat D, Durand D (2006) Gestation linked radical oxygen species fluxes and vitamins and trace mineral deficiencies in the ruminant. Reproduction Nutrition Development 46: 601–620. 10.1051/rnd:2006045 17169308

[61] Béguel J-P, Huvet A, Quillien V, Lambert C, Fabioux C (2013) Study of the antioxidant capacity in gills of the Pacific oyster *Crassostrea gigas* in link with its reproductive investment. Comparative Biochemistry and Physiology Part C: Toxicology & Pharmacology 157: 63–71. 23073513 10.1016/j.cbpc.2012.10.004

[62] Mutinati M, Piccinno M, Roncetti M, Campanile D, Rizzo A, et al (2013) Oxidative stress during pregnancy in the sheep. Reproduction in Domestic Animals. 10.1111/rda.12141 23346938

[63] Bennett NC, Jarvis JUM, Faulkes CG, Millar RP (1993) LH responses to single doses of exogenous GnRH by freshly captured Damaraland mole-rats, *Cryptomys-damarensis* . Journal of Reproduction and Fertility 99: 81–86. 8283457 10.1530/jrf.0.0990081

[64] Bennett NC (1994) Reproductive suppression in social *Cryptomys damarensis* colonies - a lifetime of socially-induced sterility in males and females (Rodentia, Bathyergidae). Journal of Zoology 234: 25–39.

[65] Bennett NC, Jarvis JUM, Millar RP, Sasano H, Ntshinga KV (1994) Reproductive suppression in eusocial *Cryptomys damarensis* colonies - socially-induced infertility in females. Journal of Zoology 233: 617–630.

[66] Bennett NC, Faulkes CG, Molteno AJ (1996) Reproductive suppression in subordinate, non-breeding female Damaraland mole-rats: Two components to a lifetime of socially induced infertility. Proceedings of the Royal Society B-Biological Sciences 263: 1599–1603. 10.1098/rspb.1996.0234 8952096

[67] Seehuus S-C, Norberg K, Gimsa U, Krekling T, Amdam GV (2006) Reproductive protein protects functionally sterile honey bee workers from oxidative stress. Proceedings of the National Academy of Sciences of the United States of America 103: 962–967. 16418279 10.1073/pnas.0502681103 PMC1347965

[68] Corona M, Velarde RA, Remolina S, Moran-Lauter A, Wang Y, et al (2007) Vitellogenin, juvenile hormone, insulin signaling, and queen honey bee longevity. Proceedings of the National Academy of Sciences of the United States of America 104: 7128–7133. 17438290 10.1073/pnas.0701909104 PMC1852330

[69] Chen JS, Sappington TW, Raikhel AS (1997) Extensive sequence conservation among insect, nematode, and vertebrate vitellogenins reveals ancient common ancestry. Journal of Molecular Evolution 44: 440–451. 9089084 10.1007/pl00006164

[70] Sappington TW, Raikhel AS (1998) Molecular characteristics of insect vitellogenins and vitellogenin receptors. Insect Biochemistry and Molecular Biology 28: 277–300. 9692232 10.1016/s0965-1748(97)00110-0

[71] Smolenaars MMW, Madsen O, Rodenburg KW, Van der Horst DJ (2007) Molecular diversity and evolution of the large lipid transfer protein superfamily. Journal of Lipid Research 48: 489–502. 17148551 10.1194/jlr.R600028-JLR200

[72] Mattson MP (2008) Hormesis defined. Aging Research Reviews 7: 1–7. 18162444 10.1016/j.arr.2007.08.007 PMC2248601

[73] Costantini D, Metcalfe NB, Monaghan P (2010) Ecological processes in a hormetic framework. Ecology Letters 13: 1435–1447. 20849442 10.1111/j.1461-0248.2010.01531.x

[74] Speakman JR, Selman C (2011) The free-radical damage theory: Accumulating evidence against a simple link of oxidative stress to aging and lifespan. Bioessays 33: 255–259. 21290398 10.1002/bies.201000132

[75] Ristow M, Schmeisser S (2011) Extending life span by increasing oxidative stress. Free Radical Biology and Medicine 51: 327–336. 21619928 10.1016/j.freeradbiomed.2011.05.010

[76] Ristow M, Zarse K (2010) How increased oxidative stress promotes longevity and metabolic health: The concept of mitochondrial hormesis (mitohormesis). Experimental Gerontology 45: 410–418. 20350594 10.1016/j.exger.2010.03.014

[77] Radak Z, Chung HY, Goto S (2008) Systemic adaptation to oxidative challenge induced by regular exercise. Free Radical Biology and Medicine 44: 153–159. 18191751 10.1016/j.freeradbiomed.2007.01.029

[78] Radak Z, Chung HY, Koltai E, Taylor AW, Goto S (2008) Exercise, oxidative stress and hormesis. Aging Research Reviews 7: 34–42. 17869589 10.1016/j.arr.2007.04.004

[79] Lovegrove BG (1987) The energetics of sociality in mole-rats (Bathyergidae) Cape Town: University of Cape Town.

[80] Bennett NC (1990) Behavior and social-organization in a colony of the Damaraland mole-rat *Cryptomys-damarensis* . Journal of Zoology 220: 225–248.

[81] Perez-Campo R, Lopez-Torres M, Rojas C, Cadenas S, Barja G (1993) A comparative-study of free-radicals in vertebrates. 1. Antioxidant enzymes. Comparative Biochemistry and Physiology B-Biochemistry & Molecular Biology 105: 749–755. 10.1016/0305-0491(93)90116-m 8395990

[82] Greenberg JA (1999) Organ metabolic rates and aging: two hypotheses. Medical Hypotheses 52: 15–22. 10342665 10.1054/mehy.1997.0619

[83] Wang ZM, O'Connor TP, Heshka S, Heymsfield SB (2001) The reconstruction of Kleiber's law at the organ-tissue level. Journal of Nutrition 131: 2967–2970. 11694627 10.1093/jn/131.11.2967

[84] Kwong LK, Sohal RS (2000) Age-related changes in activities of mitochondrial electron transport complexes in various tissues of the mouse. Archives of Biochemistry and Biophysics 373: 16–22. 10620319 10.1006/abbi.1999.1495

[85] Metcalfe NB, Monaghan P (2013) Does reproduction cause oxidative stress? An open question. Trends in Ecology & Evolution 28: 347–350. 23485157 10.1016/j.tree.2013.01.015

[86] Page RE, Peng CYS (2001) Aging and development in social insects with emphasis on the honey bee, *Apis mellifera* . Experimental Gerontology 36: 695–711. 11295509 10.1016/s0531-5565(00)00236-9

[87] Schrempf A, Cremer S, Heinze J (2011) Social influence on age and reproduction: reduced lifespan and fecundity in multi-queen ant colonies. Journal of Evolutionary Biology 24: 1455–1461. 21507120 10.1111/j.1420-9101.2011.02278.x

[88] Schrempf A, Heinze J, Cremer S (2005) Sexual cooperation: Mating increases longevity in ant queens. Current Biology 15: 267–270. 15694312 10.1016/j.cub.2005.01.036

