# Supplementary material for: Reproduction Is Associated with a Tissue-Dependent Reduction of Oxidative Stress in Eusocial Female Damaraland Mole-Rats (Fukomys damarensis)
Source: PLoS One. 2014 Jul 28;9(7):e103286. doi: 10.1371/journal.pone.0103286 (PMC4113376; doi:10.1371/journal.pone.0103286)
Supplement: Table S1 — Data used for the analyses of markers of oxidative damage and antioxidant defence in various tissues in relation to reproductive state. (DOCX) [file pone.0103286.s001.docx]

Reproduction is associated with a tissue-dependent reduction of oxidative stress in eusocial female Damaraland mole-rats (*Fukomys damarensis*)

Christina M Schmidt, Jonathan D Blount, and Nigel C Bennett

Table S1. Data used for the analyses of markers of oxidative damage and antioxidant defence in various tissues in relation to reproductive state.

| Marker | Tissue | Reproductive state | Mean | s.d. | N |
| --- | --- | --- | --- | --- | --- |
| MDA | Heart | Reproductive | 7.43 | 1.57 | 9 |
|  |  | Non-reproductive | 11.60 | 6.14 | 12 |
|  | Kidney | Reproductive | 46.84 | 19.74 | 9 |
|  |  | Non-reproductive | 68.59 | 31.73 | 14 |
|  | Liver | Reproductive | 26.16 | 7.31 | 9 |
|  |  | Non-reproductive | 64.74 | 26.00 | 14 |
|  | Skeletal muscle | Reproductive | 23.23 | 10.11 | 7 |
|  |  | Non-reproductive | 25.72 | 15.75 | 14 |
|  | Plasma | Reproductive | 1.68 | 1.06 | 9 |
|  |  | Non-reproductive | 1.18 | 0.44 | 14 |
| PC | Heart | Reproductive | 2.07 | 1.45 | 6 |
|  |  | Non-reproductive | 4.67 | 3.90 | 7 |
|  | Kidney | Reproductive | 1.83 | 1.44 | 7 |
|  |  | Non-reproductive | 4.05 | 1.76 | 12 |
|  | Liver | Reproductive | 3.05 | 0.97 | 9 |
|  |  | Non-reproductive | 4.23 | 3.10 | 12 |
|  | Skeletal muscle | Reproductive | 3.16 | 1.74 | 4 |
|  |  | Non-reproductive | 4.34 | 2.93 | 8 |
|  | Plasma | Reproductive | 1.85 | 0.54 | 9 |
|  |  | Non-reproductive | 2.12 | 1.28 | 11 |
| TAC | Heart | Reproductive | 0.26 | 0.06 | 9 |
|  |  | Non-reproductive | 0.32 | 0.07 | 12 |
|  | Kidney | Reproductive | 7.05 | 2.17 | 9 |
|  |  | Non-reproductive | 6.22 | 1.67 | 14 |
|  | Liver | Reproductive | 18.54 | 4.08 | 8 |
|  |  | Non-reproductive | 18.42 | 3.63 | 14 |
|  | Plasma | Reproductive | 1.11 | 0.74 | 9 |
|  |  | Non-reproductive | 0.95 | 0.58 | 12 |
| SOD | Heart | Reproductive | 0.95 | 0.45 | 9 |
|  |  | Non-reproductive | 0.86 | 0.34 | 12 |
|  | Kidney | Reproductive | 2.72 | 1.00 | 9 |
|  |  | Non-reproductive | 3.05 | 0.65 | 14 |
|  | Liver | Reproductive | 5.04 | 2.97 | 9 |
|  |  | Non-reproductive | 3.87 | 2.07 | 14 |
|  | Skeletal muscle | Reproductive | 0.76 | 0.45 | 8 |
|  |  | Non-reproductive | 0.35 | 0.07 | 6 |
|  | Erythrocytes | Reproductive | 594.96 | 426.90 | 8 |
|  |  | Non-reproductive | 793.36 | 352.14 | 9 |
